# Rs12976445 polymorphism is associated with the risk of post-SAH re-bleeding by modulating the expression of microRNA-125 and ET-1

**DOI:** 10.1038/s41598-021-04330-4

**Published:** 2022-02-08

**Authors:** Wenping Xiong, Weiqi Yao, Zeyuan Gao, Kui Liu

**Affiliations:** 1 Department of Neurosurgery, Zhongnan Hospital of Wuhan University, No. 169 Donghu Road, Wuhan, 430071 China; 2 Department of Hematology, Union Hospital, Huazhong University of Science and Technology, Hubei Engineering Research Center for Human Stem Cell Preparation and Application and Resource Conservation, Wuhan, 430022 China

**Keywords:** Cell biology, Molecular biology

## Abstract

This study aimed to study the association between rs12976445 polymorphism and the incidence of IA re-bleeding. Genotype and allele frequency analysis was performed to study the association between rs12976445 polymorphism and the risk of IA re-bleeding. Western blot, ELISA and real-time RT-PCR were conducted to measure the relative expression of miR-125a, ET1 mRNA and ET1 protein. Computational analysis and luciferase assays were utilized to investigate the association between the expression of miR-125a and ET1 mRNA. No significant differences were observed between IA patients with or without symptoms of re-bleeding. Subsequent analyses indicated that the T allele was significantly associated with the reduced risk of IA re-bleeding. In patients carrying the CC genotype, miR-125a level was up-regulated while ET1 mRNA/protein levels were reduced compared with those in patients carrying the CT or TT genotype. And ET1 mRNA was identified as a virtual target gene of miR-125a with a potential miR-125a binding site located on its 3’UTR. Accordingly, the ET mRNA/protein levels could be suppressed by the transfection of miR-125a precursors, but the transfection of ET1 siRNA exhibited no effect on the expression of miR-125a. Therefore, an increased level of miR-125a can lead to the increased risk of IA re-bleeding. Since miR-125a level is higher in CC-genotyped patients, it can be concluded that the presence of T allele in the rs12976445 polymorphism is associated with a lower risk of IA re-bleeding, and miR-125a may be used as a novel diagnostic and therapeutic target for IA rupture.

## Introduction

Frequently developed at the branch of cerebral arteries at the bottom portion of brain, intracranial aneurysms (IA) impairs the inherent structure in the wall of cerebral arteries without disrupting the structure of elastic lamina or inducing hemodynamic stresses^1–4^. However, the rupture of the aneurysm may cause subarachnoid hemorrhage (SAH), which in turn poses a greater risk to IA patients^5^. Moreover, the incidence of SAH can be up to 50% in IA patients, while over 70% of SAH patients have a poor prognosis^5–8^. And past investigations have shown that IA patients experiencing repeated aneurysm bleeding are challenged by reduced brain functions^9^. Therefore, it is recommended to control the blood pressure and apply anti-fibrinolytic treatments in IA patients^9^.

Endothelin-1 (ET-1) was discovered nearly two decades ago as a compound with important roles in the development of multiple cardiovascular diseases^10–12^. Released from impaired endothelia, ET-1 may be utilized as a new target in the treatment of a broad range of cardiovascular diseases. For example, agents have been developed to modulate the activity of ET-1 in various cardiovascular diseases, as well as to block the synthesis of ET-1^13,14^. In particular, the implication of ET-1 in ischemic heart diseases, hypertension, congestive heart failures, cerebral vasospasm, Raynaud’s syndrome, and SAH has been confirmed in patients with an increased level of vessel tone^15^. Furthermore, some studies demonstrated the involvement of ET receptors in the SAH of the aneurysm. In contrary, a preliminary study showed no effects of BMS-193884, an antagonist of ETA, in the treatment of erectile dysfunction in 53 patients^14^.

Usually 21 to 24 nt long, microRNAs (miRNAs) can bind to the 3′‐UTR of their target mRNAs and subsequently repress mRNA expression. In fact, miRNAs play essential roles in many physiological activities such as cell cycle progression, differentiation of stem cells, organ growth and signal transduction^16^. MiRNAs have also been implicated in tumorigenesis and the regulation of body response upon oxidative stress, DNA damages, and irradiation^17^. For example, miR‐125 was shown to regulate innate immune responses in Drosophila^18^. Furthermore, miR‐125a can decrease inflammatory cytokine synthesis and lipid intake in macrophages upon the stimulation of low density lipoprotein^19^. Moreover, miR‐125a can reduce the amount of surface antigen of hepatitis B virus while inducing M2 macrophage polarization by decreasing M1 macrophage polarization^20,21^. Moreover, miR‐125a also participates in immune regulation by increasing its expression in CD4 + CD25 − T cells^22^.

Single nucleotide polymorphism greatly affects the expression of its host genes. In particular, SNP rs12976445 found in the sequence of miR‐125a could reduce its expression in several diseases^23^. In fact, the impaired functions of TGFβ1 were shown to increase the risk of pneumonitis induced by RTI^24^. In this study, we collected samples from IA patients with or without symptoms of re-bleeding and studied the association between the rs12976445 polymorphism and the risk of post-SAH re-bleeding.

## Materials and methods

### Human IA patients and sample collection

SAH patients with (N = 182) or without (N = 386) re-bleeding were enrolled in this study as case and control groups, respectively. The demographic data of all participants were collected to compare the differences between the two groups (Table 1). According to their genotypes of the rs12976445 SNP, the subjects in case and control groups were further divided into different genotype groups, i.e., CC, CT and TT groups for the subsequent analysis of genotype frequency and association. Furthermore, 50 IA patients who have received surgical intervention were randomly selected from the recruited SAH patients with re-bleeding for the subsequent genotype frequency and association analysis. Accordingly, they were genotyped and divided into the CC group (N = 28), CT group (N = 16) and TT group (N = 6). This study was approved by the medical ethics committee of Zhongnan Hospital of Wuhan University and all subjects have provided a signed form of informed consent before participating in the study. All methods were performed in accordance with the last vision of the Declaration of Helsinki.

**Table 1:** Demographic data of the subjects with and without re-bleeding.

	SAH ( +) Re-bleeding (-)	SAH ( +) Re-bleeding ( +)	*P* value
Number	386	182	
Male (%)	239 (62%)	118 (65%)	
Female (%)	147 (38%)	64 (35%)	0.873
Hypertension (%)	101 (26%)	53 (29%)	0.212
Smoker (%)	81 (21%)	46 (26%)	0.123
Drinker (%)	73 (19%)	43 (24%)	0.097

### Genotyping of rs12976445 SNP in miR-125a by Taqman assays

The genomic DNA was isolated from the plasma samples collected from all subjects by a Quick Serum DNA Purification Assay Kit (Thermo Fisher Scientific, Waltham, MA). In the next step, the concentration of genomic DNA in each sample was adjusted to about 250 mg/ml by measuring the DNA concentration in each sample with a Nano Drop 3000 spectrophotometer. Subsequently, the isolated genomic DNA was used as the template to generate amplified DNA using real-time PCR. Finally, the genotypes of rs12976445 SNP were determined by a TaqMan genotyping assay using real-time PCR performed on an ABI 7900 real-time PCR machine (ABI, Foster City, CA).

### RNA isolation and real-time polymerase chain reaction (PCR) of miR-125a and ET1 mRNA

Both plasma and aneurysm tissue samples were defrosted to extract their RNA content using a High Yield RNA Purification Kit (Roche, New York City, NY). In the next step, a reaction of reverse transcription was carried out with universal primers to convert isolated RNA samples into cDNA templates. The volume of each sample for RT reaction was 25 μl containing a SuperScript Mix and 5 μl of RNA template. In the next step, the cDNA templates were subject to real-time PCR with a Platinum Taq Enzyme (Invitrogen, Carlsbad, CA) in conjunction with a HiScript II One-Step SYBR Green assay kit (Biotime, Nanjing, China). The volume in each well of the real-time PCR reaction plate was 20 μl, which contained both forward and reverse primers of miR-125a and ET1 mRNA, respectively. The program of real-time PCR reaction included 5 min of Taq polymerase activation at 95 °C and then 45 cycles of real-time PCR reactions performed for 10 s at 95 °C and 30 s for 60 °C in each cycle. Finally, the relative expression of miR-125a (Forward: 5’-CCTGAGACCCTTTAACC-3’; Reverse: 5’-GAACATGTCTGCGTATCTC-3’) and ET1 mRNA (Forward: 5’-CTACTTCTGCCACCTGGACATC-3’; Reverse: 5’- TCACGGTCTGTTGCCTTTGTGG3’) was quantified using the cycle threshold values^25^, and the expression of U6 (Forward: 5’-GTGCTCGCTTCGGCAGCA-3’; Reverse: 5’-CAAAATATGGAACGCTTC-3’) and GAPDH (Forward: 5’-GAAGGTGAAGGTCGGAGTC-3’; Reverse: 5’-GAAGATGGTGATGGGATTTC-3’) respectively was used as the internal controls.

### Culture of SH-SY5Y and U251 cells and transfection

SH-SY5Y and U251 cells (ATCC, Manassas, VA, US) were maintained on tissue culture dishes coated with type I collagens (Nitta Gelatin, Tokyo, Japan) and grown in low glucose DMEM (Wako, Tokyo, Japan) added with 10% fetal bovine serum (Thermo Fisher Scientific, Tokyo, Japan) and appropriate antibiotics (Wako, Tokyo, Japan). For transfection, SH-SY5Y and U251 cells were inoculated into 24-well tissue culture plates and then transfected with NC, miR-125a precursor, or ET1 siRNA by Fugene 6 HD (Promega, Madison, WI). At 48 h after the transfection, transfected cells were harvested to assay the expression levels of ET1. Data were averaged from 3 repeated experiments.

### Vector construction, mutagenesis and luciferase assay

As reported previously, the 3’ UTR of ET1 contains a binding site for miR-125a. In this study, the 3’ UTR of ET1 promoter containing the binding site for miR-125a was sub-cloned into a luciferase pcDNA vector (Promega, Madison, WI), which was termed the vector of wild type ET1 3’ UTR. After the correct sequence of the wild type ET1 3’ UTR vector was validated by direct sequencing, site-directed mutagenesis was performed at the miR-125a binding site located in the 3’ UTR of ET1, and the mutant 3’ UTR of ET1 was also sub-cloned into a luciferase pcDNA vector and termed the mutant ET1 3’ UTR vector. In the next step, SH-SY5Y and U251 cells were inoculated into 24-well tissue culture plates and then transfected with miR-125a and wild type/mutant 3’ UTR of ET1 using Fugene 6 HD. At 48 h after the transfection, the luciferase activity of transfected cells in each well was assayed using Promega’s Dual luciferase assay kit. The expression levels of ET1 were obtained from the average of 3 repeated experiments.

### Western blot analysis

The protein expression of ET1 in cultured cell samples and clinical samples were measured by Western blot analysis. In brief, lysates of samples were prepared by tissue/cell homogenization and a subsequent short treatment of sonication in the lysis buffer containing 50 mM of Tris, 0.1% of CHAPS, 150 mM of dithiothreitol, 150 mM of NaCl and a cocktail of protease inhibitors (pH 7.4) (Roche, New York City, NY). The protein concentration in the lysates was confirmed with a Quick Bradford Protein Assay (Bio-Rad Laboratories, Hercules, CA). In the next step, the lysates obtained from different samples were separated on a 10% polyacrylamide gel by running the gel for 1 h at 200 V. Then, the protein blots were transferred at 400 mA to a PVDF membrane (GE, Chicago, IL) under soaking in a transfer buffer of sodium borate for 70 min. In the next step, the membrane was blocked using TBST (pH 7.4, containing 10 mM of Tris, 0.1% of Tween-80, and 150 mM of NaCl) containing 5% skim milk, and then incubated at 4 °C overnight with anti-ET1 and anti-β antibodies (internal control, Abcam, Cambridge, MA). After being incubated with HRP-IgG secondary antibodies and developed by an ECL reagent (Sycamore, Houston, TX), the relative expression of ET1 was calculated using Image J software.

### Enzyme linked immunosorbent assay (ELISA)

An ELISA assay was carried out to detect the levels of ET1 protein in tissue and cell samples using a commercially available kit (Proteintech, Wuhan, China) following the kit instruction.

### Statistical analysis

The analysis of genotype and allele frequency in collected samples was carried out using the Hardy–Weinberg equilibrium test in conjunction with Fisher’s exact and Chi-squared tests. The distributions of genotype and allele frequency in the samples were analyzed using the Kruskal–Wallis test. The correlation between different genotypes and the risk of IA was obtained using Pearson correlation analysis and logistic regression analysis. The difference between multiple groups was compared by one-way ANOVA followed by Tukey’s test. All data was expressed using mean ± standard deviations. All statistical analyses were carried out using Prism 7.0 (GraphPad, San Diego, CA) and SPSS 21.0 (IBM, Chicago, IL). *P* < 0.05 was considered significant for statistical comparisons.

## Results

### Rs12976445 polymorphism was associated with the risk of IA re-bleeding

Patients with IA re-bleeding (N = 182) were recruited as the case group in this study, with 368 IA patient showing no symptoms of re-bleeding being recruited as the control group. The demographic data of all participants was collected to compare the differences between the two groups. As shown in Table 1, there were no evident differences between case and control groups. According to their genotypes of the rs12976445 SNP, the subjects in the case group were further divided into a CC group (N = 80), a CT group (N = 82) and a TT group (N = 20), while the subjects in the control group were also divided into a CC group (N = 263), a CT group (N = 109) and a TT group (N = 14). The frequency of each genotype and allele of rs12976445 polymorphism was presented in Table 2. According to Table 2, the heterozygous genotype CT was borderline associated with a lowered risk of IA re-bleeding, while the homozygous genotype CC showed the highest risk of IA re-bleeding. Additionally, unlike the C allele, the T allele was significantly associated with a reduced risk of IA re-bleeding.

**Table 2:** Comparison of genotype and allele frequency between the subjects with and without re-bleeding.

	SAH ( +) Re-bleeding (-)	SAH ( +) Re-bleeding ( +)	OR (95%CI)	*P* value
CC	263 (68.23%)	80 (30.35%)	Reference	
CT	109 (28.45%)	82 (45.65%)	2.47 (1.69–3.61)	< 0.0001
TT	14 (3.32%)	20 (25.00%)	4.29 (2.26–9.72)	< 0.0001
CT/TT	213 (31.79%)	102(70.65%)	1.57 (1.11–2.22)	< 0.01
C	635 (82.25%)	242 (66.48%)	Reference	
T	137 (17.75%)	122 (33.52%)	2.33 (1.75–3.10)	< 0.0001

### MiR-125a and ET1 mRNA/protein were differently expressed in patients carrying different genotypes of rs12976445 polymorphism

**Figure 1:**
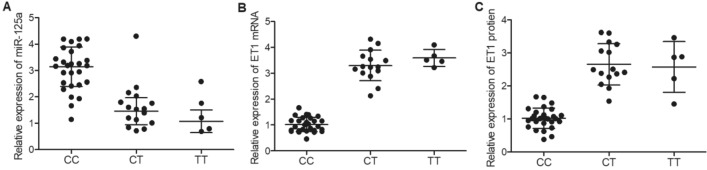
MiR-125a and ET1 mRNA/protein were differently expressed in IA tissue samples collected from IA re-bleeding patients carrying different genotypes of rs12976445 polymorphism. (**A**) Relative expression of miR-125a was evidently lower in the CT and TT groups compared with that in the CC genotype group; (**B**) Relative expression of ET1 mRNA was evidently increased in patients carrying the CT and TT genotypes compared with that in patients carrying the CC genotype; (**C**) ET1 protein was highly expressed in patients carrying the CT and TT genotypes compared with that in patients carrying the CC genotype.

**Figure 2:**
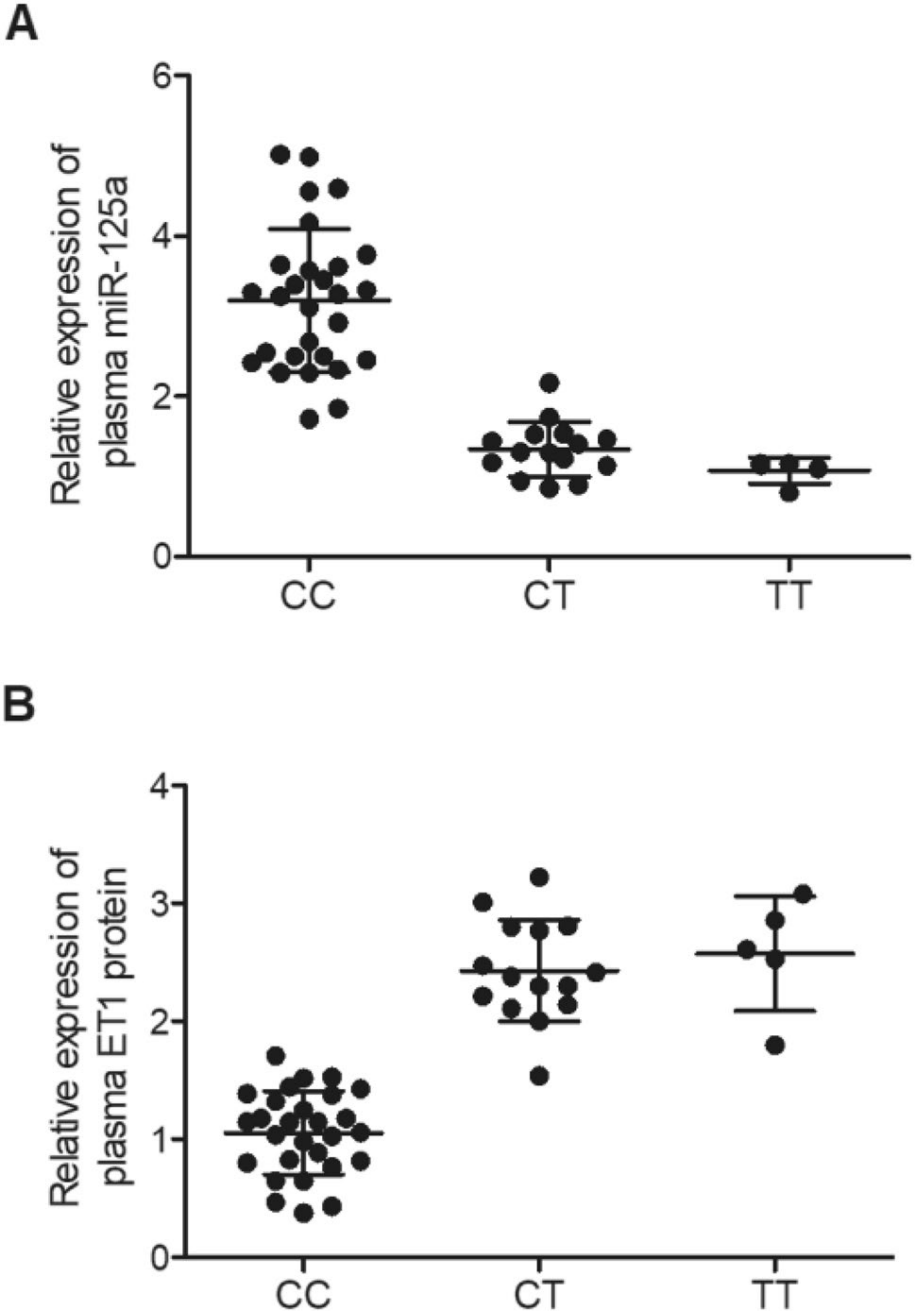
Plasma miR-125a and ET1 protein expression was different among IA re-bleeding patients carrying different genotypes. (**A**) Relative expression of plasma miR-125a was decreased in patients carrying the CT and TT genotypes compared with that in patients carrying the CC genotype; (**B**) Relative expression of plasma ET1 protein was evidently increased in patients carrying the CT and TT genotypes compared with that in patients carrying the CC genotype.

In order to understand how rs12976445 polymorphism is associated with the risk of IA re-bleeding, aneurysm tissue and plasma samples were collected from the 50 IA patients, who were genotyped as the CC group (N = 28), CT group (N = 16) and TT group (N = 6), for further analysis. By conducting qRT-PCR and Western Blot assays on the aneurysm tissue samples collected from patients carrying different rs12976445 genotypes, it was found that the relative expression of miR-155 was evidently up-regulated in the CC group (Fig. 1A). However, the relative expression of ET1 mRNA (Fig. 1B) and protein (Fig. 1C) was significantly down-regulated in the CC group. Similarly, plasma levels of miR-125a (Fig. 2A) and ET1 protein (Fig. 2B) both exhibited similar trends as those in tissue samples.

### Expression of miR-125a was negatively correlated with the expression of ET1 mRNA/protein

**Figure 3:**
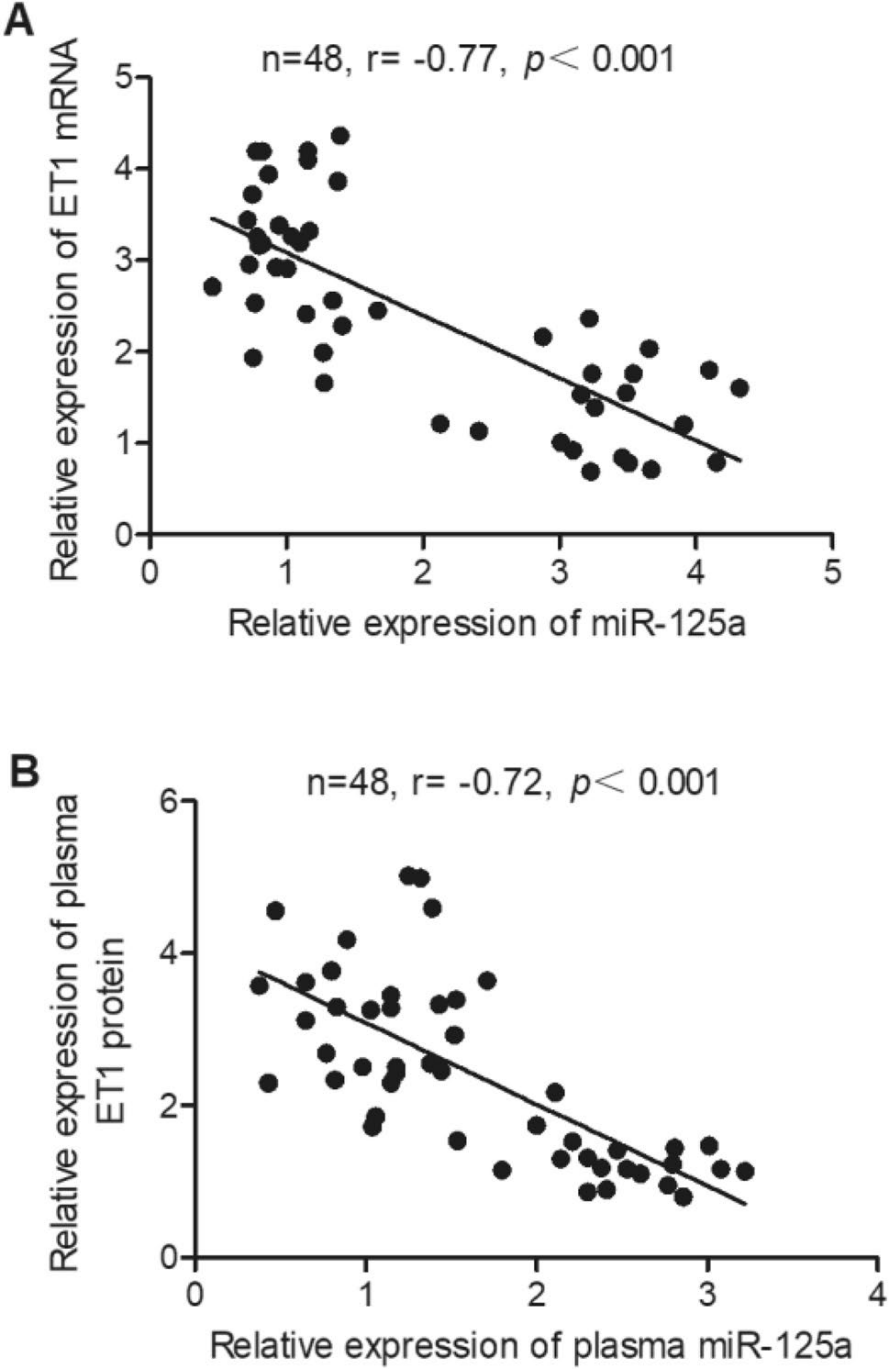
The expression of miR-125a was negatively correlated with the expression of ET1 mRNA/protein. (**A**) Linear correlation analysis showed a negative correlation between the relative expression of miR-125a and that of ET1 mRNA in IA tissue samples; (**B**) Linear correlation analysis showed a negative correlation between the relative expression of plasma miR-125a and that of plasma ET1 protein in plasma samples.

To clarify the possible association between the expression of miR-125a and ET1 mRNA/protein, a linear correlation analysis was performed using collected aneurysm tissue and plasma samples. As shown in Fig. 3A, a negative correlation was observed between miR-125a expression and ET1 mRNA expression in aneurysm tissue samples. In addition, as shown in Fig. 3B, the expression of miR-125a was also negatively correlated with the expression of ET protein in plasma samples. Therefore, we conclude that the expression of ET mRNA/protein is modulated by miR-125a.

### ET1 mRNA was a virtual target gene of miR-125a

**Figure 4:**
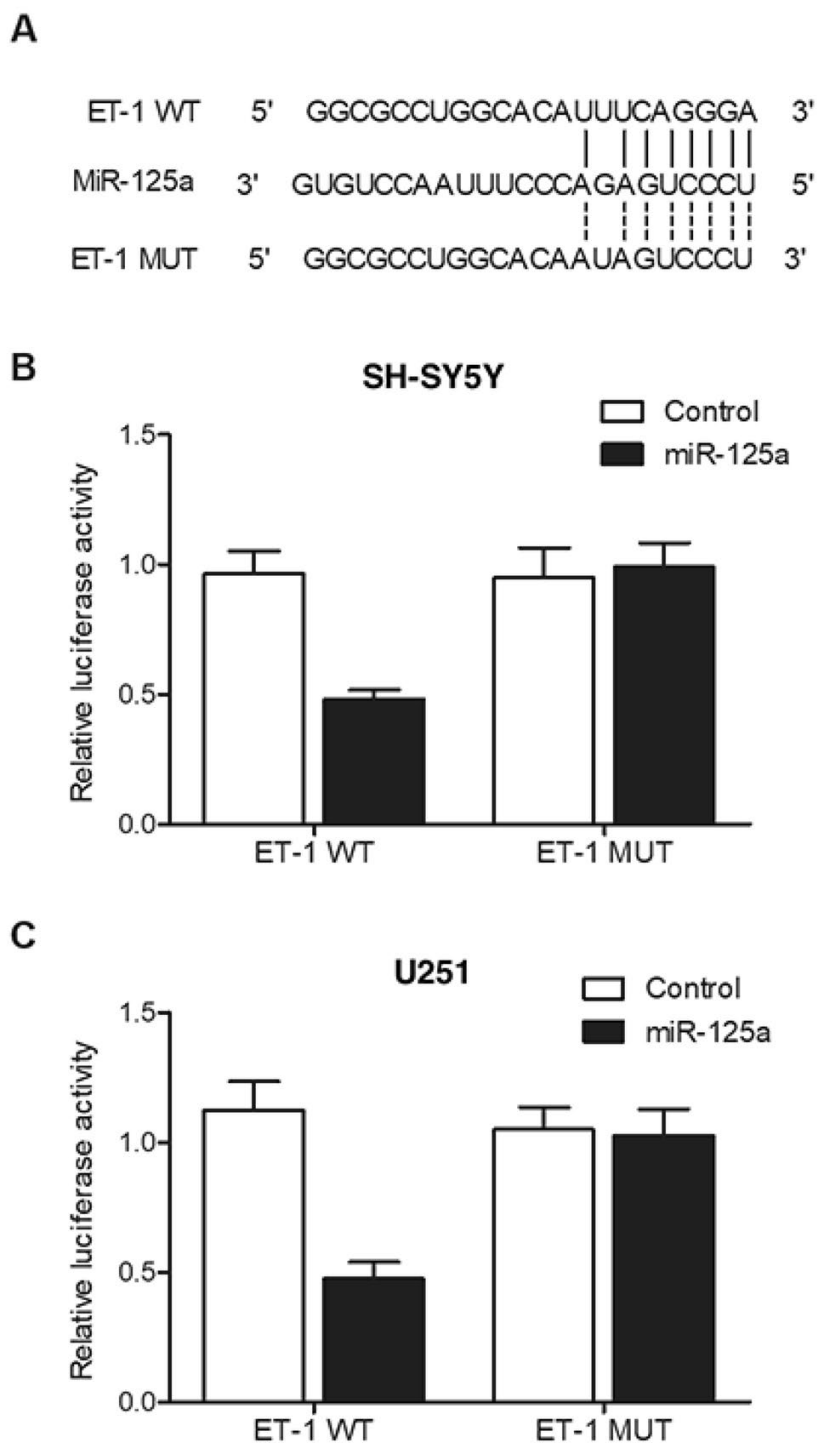
ET1 mRNA was identified as a virtual target gene of miR-125a. (**A**) A miR-125a binding site was located on the 3’UTR of ET1 mRNA; (**B**) Relative luciferase activity of wild-type ET1 mRNA was evidently reduced in SH-SY5Y cells transfected with miR-125a; (**C**) Relative luciferase activity of wild-type ET1 mRNA was evidently reduced in U251 cells transfected with miR-125a.

Based on the results from literature research and computational analyses using online miRNA predicting tools, a potential miR-125a binding site was found on the 3’UTR of ET1 mRNA (Fig. 4A). A luciferase assay was then utilized to further investigate the relationship between the expression of ET1 mRNA and miR-125a by transfecting SH-SY5Y and U251 cells with miR-125a or negative controls. The luciferase assays showed that only the luciferase activity of wild-type ET1 mRNA was significantly suppressed by miR-125a in SH-SY5Y (Fig. 4B) and U251 cells (Fig. 4C), indicating that ET1 mRNA was a virtual target gene of miR-125a.

### MiR-125a negatively regulated the expression of ET1 mRNA

**Figure 5:**
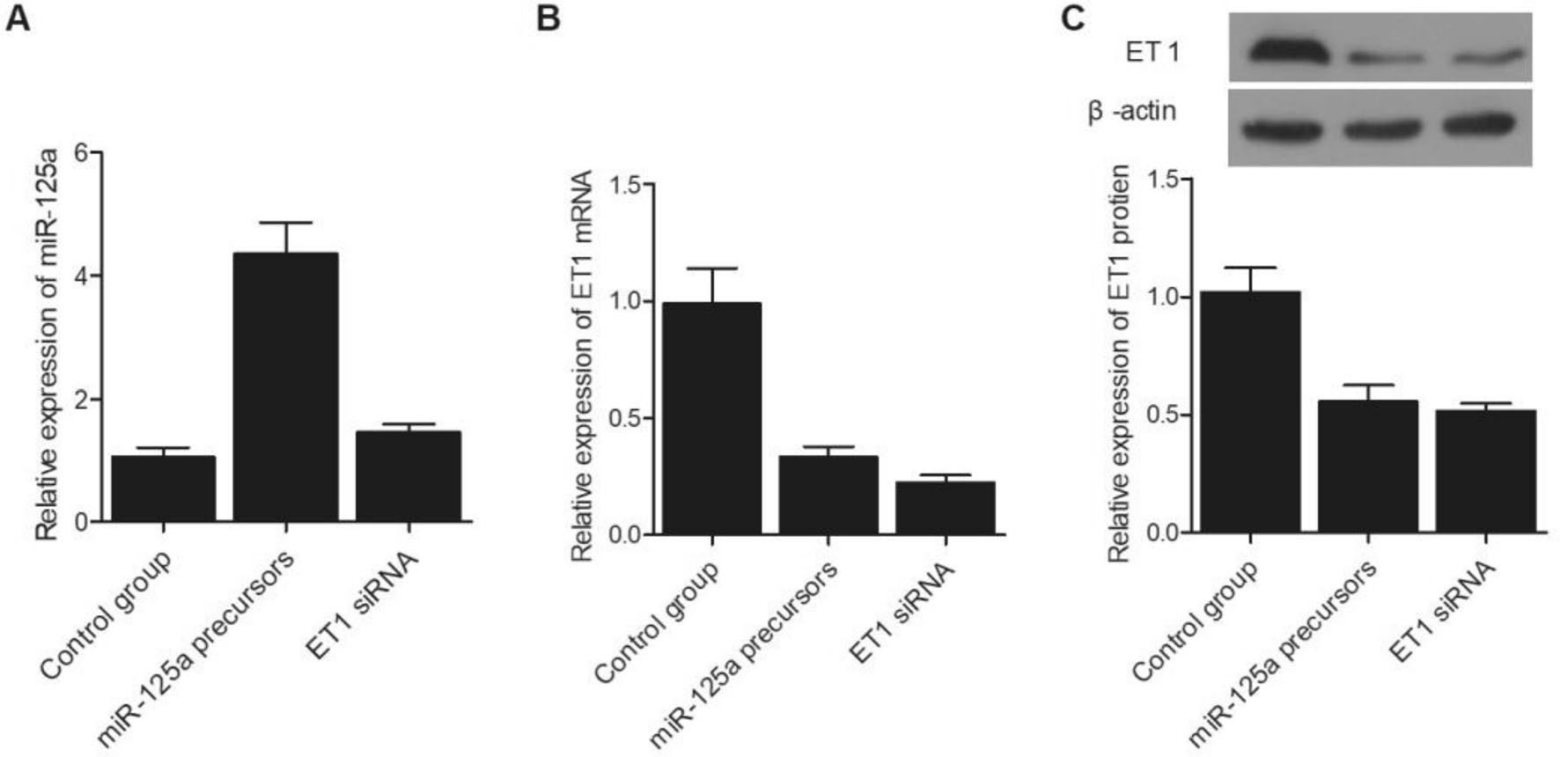
Expression of miR-125a and ET1 mRNA/protein in SH-SY5Y cells was influenced by the transfection of miR-125a precursors or ET1 siRNA. (**A**) Relative expression of miR-125a in SH-SY5Y cells was elevated after the transfection with miR-125a precursors, while the transfection of ET1 siRNA exhibited no evident effect on the expression of miR-125a; (**B**) Relative expression of ET1 mRNA in SH-SY5Y cells was evidently suppressed after the transfection with miR-125a precursors or ET1 siRNA; (**C**) Relative expression of ET1 protein in SH-SY5Y cells was significantly lower after the transfection with miR-125a precursors or ET1 siRNA.

**Figure 6:**
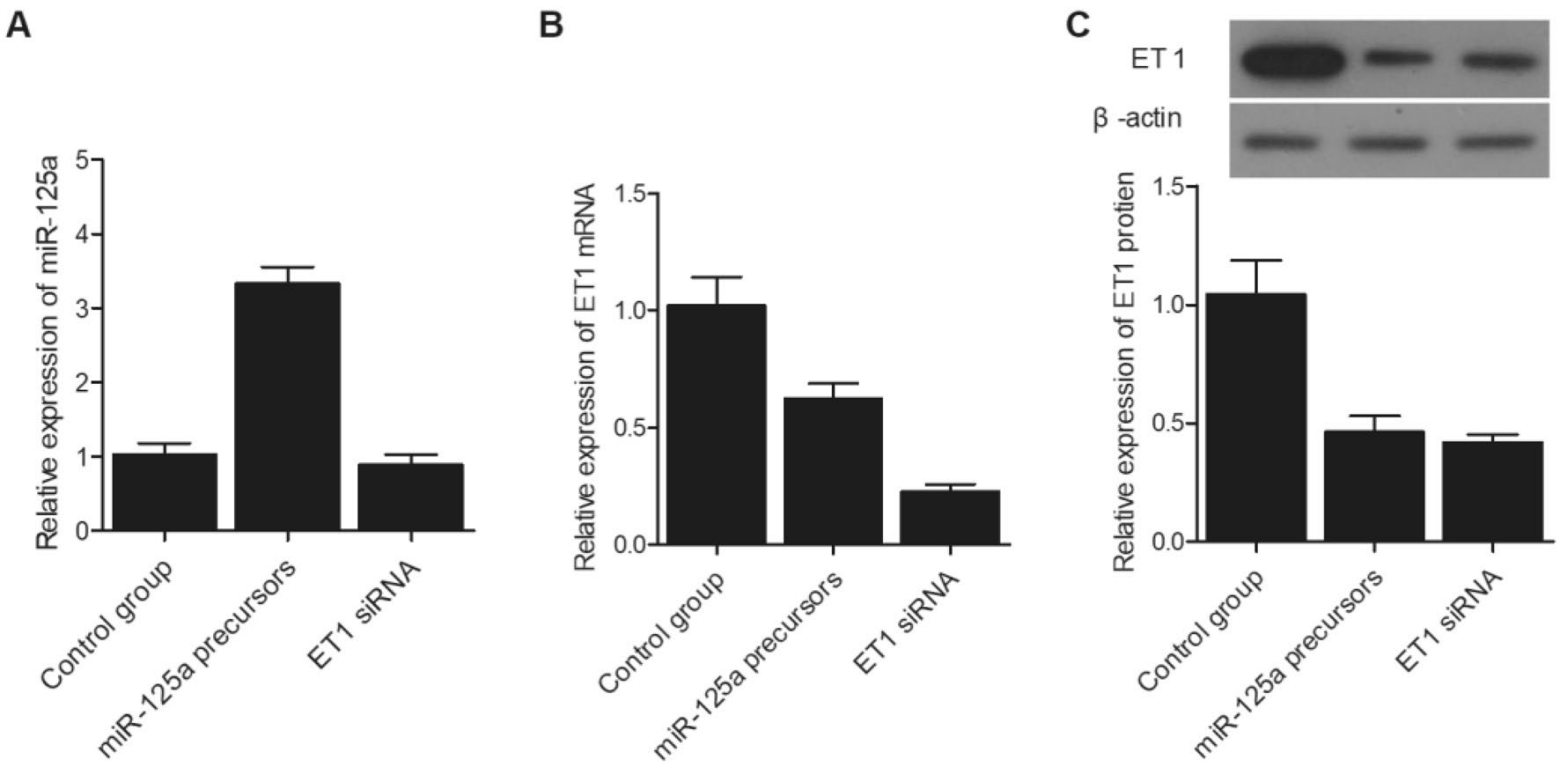
Expression of miR-125a and ET1 mRNA/protein in U251 cells was influenced by the transfection of miR-125a precursors or ET1 siRNA. (**A**) Relative expression of miR-125a in U251 cells was elevated after the transfection with miR-125a precursors, while the transfection of ET1 siRNA exhibited no evident effect on the expression of miR-125a; (**B**) Relative expression of ET1 mRNA in U251 cells was evidently suppressed after the transfection with miR-125a precursors or ET1 siRNA; (**C**) Relative expression of ET1 protein in U251 cells was significantly lower after the transfection with miR-125a precursors or ET1 siRNA.

SH-SY5Y cells were divided into three different groups and transfected with miR-125a precursors, ET1 siRNA, and a control miRNA, respectively. As shown in Fig. 5A, the relative expression of miR-125a was similar between the control group and ET siRNA group, while the transfection of miR-125a precursors evidently enhanced the expression of miR-125a in SH-SY5Y cells. Meanwhile, compared with that in the control group, the expression of ET1 mRNA (Fig. 5B) and protein (Fig. 5C) was both suppressed by the transfection of ET1 siRNA and miR-125a precursors. Similar results were obtained in U251 cells, indicating that an increased level of miR-125a could lead to reduced ET1 protein expression and an increased risk of IA re-bleeding (Fig. 6). Since miR-125a level is higher in patients carrying the CC genotype of rs12976445 polymorphism, it can be concluded that the T allele in rs12976445 polymorphism is associated with a lower risk of IA re-bleeding.

## Discussion

The SAH in the aneurysm, i.e., aSAH, is a special type of SAH caused by the damage to the cerebral aneurysm. Due to its site of damage, aSAH is associated with increased levels of mortality and morbidity. In addition, the incidence rates of aSAH in different countries are considerably different, from as low as 2 cases in every 100,000 people to as high as 16 cases in every 100,000 people^26^. Repeated bleeding of the cerebral aneurysm during aSAH significantly deteriorates the prognosis of patients and increases their mortality^27^. For example, the mortality of aSAH patients suffering from early episodes of bleeding can be up to 15% in the first few days^27,28^.

MiR-125a was shown to exert important effects on the tissue growth in both adults and newborns^29–31^. Furthermore, it was demonstrated that miR-125a contributes to the onset of cancers, such as lung, breast, and colon cancers^32–35^. In fact, miR-125a can act either as a tumor inhibitor or a tumor promoter under different circumstances. In certain cancers such as lung cancer, gastric cancer, breast cancer, medulloblastoma, and leukemia, the expression of miR-125a was decreased and an increased level of miR-125a expression in these cancers inhibited the proliferation and metastasis of cancer^32–37^. Moreover, p53 was found to mediate the function of miR-125a in the proliferation and metastasis of myeloma^31^. According to computational analysis and subsequent luciferase assays, ET1 mRNA was identified as a virtual target gene of miR-125a. The expression of ET mRNA/protein could be suppressed by the transfection of miR-125a precursors while the transfection of ET1 siRNA exhibited no effect on the expression of miR-125a. Therefore, an increased level of miR-125a could lead to reduced ET1 protein expression and an increased risk of IA re-bleeding.

As a crucial vasoconstrictor, ET-1 participates in the regulation of post-aSAH cerebral vasospasm^38–40^. It was shown that an increased level of ET-1 synthesis in the ECs of damaged AVM and aneurysm helped to induce the formation of blood clots upon the secretion of oxyhemoglobin in the subarachnoid space. Furthermore, oxyhemoglobin can promote the vasoconstrictive effect of ET-1 by binding to the abluminal end of cerebral endothelium^41^. On the other hand, Pluta et al. failed to show that the formation of ET-1 is promoted by oxyhemoglobin^42^. Furthermore, in endothelial cells cultured in vitro and exposed to hemoglobin, the formation of ET-1 was not increased even after 6 h of hemoglobin exposure. On the other hand, the culture of astrocytes under similar conditions substantially increased the production of ET-1, suggesting that the role of oxyhemoglobin depends on the types of cells. Some other studies have also suggested that the level of ET-1 in the CSF may not be related to the stimulation by mononuclear leucocytes^43,44^. In addition, a previous study has demonstrated that the gene expression of ppET-1 was inhibited in lesions of AVM^45^. In aSAH, the production of ET is also enhanced by multiple factors, such as TGF–1^33^, oxyhemoglobin^27,28,37^ and thrombin^30,46,47^. Furthermore, if an excessive amount of subarachnoid blood cannot be removed timely, a higher level of ET–1 in the CSF can occur after endovascular treatments, although it remains unclear whether the application of acute-phase operations can lead to an increased level of ET–1 in the CSF. Because the pathogenesis of vasospasm is complex and involves many different factors, the specific role of ET in the pathogenesis of vasospasm remains unclear, although surgeries were shown to lead to a decreased level of ET–1 in the CSF. Moreover, the clinical prognosis and incidence of stroke may not be affected by surgeries carried out in the early stage of aSAH, although they may reduce the concentration of ET–1 in the CSF.

Past studies have demonstrated that the gene of pre-miR-125a carries an rs12976445 SNP implicated in the maturation of miR-125a, and the presence of this SNP increases the probability of autoimmune diseases of the thyroid and repeated pregnancy loss^48–50^. Moreover, rs12976445 leads to a poor prognosis of esophageal squamous cell cancer, whereas a past report demonstrated that the level of mature miR-125a is reduced by rs12976445 G- > T, thus reducing the inhibitory effects of miR-125a on its downstream targets, such as ERBB2 and LIFR^48–50^. Past studies also showed an essential function of ET-1 in regulating the onset of ischemic stroke-induced dementia. Furthermore, the T allele of rs12976445 SNP decreases ET-1 expression in the endothelium, resulting in an elevated risk of post-stroke cognitive disorders. Finally, the rs12976445 SNP also acts as a prognosis biomarker for stroke-induced dementia^51^. In this study, the analysis of genotype and allele frequency of rs12976445 polymorphism indicated that the genotype CC showed the highest risk of IA re-bleeding. Additionally, the T allele was significantly associated with a reduced risk of IA re-bleeding. In addition, miR-125a level was up-regulated while ET1 mRNA/protein expression was down-regulated in the aneurysm tissue and plasma samples collected from the IA re-bleeding patients in the CC group, suggesting that a negative correlation is present between the expression of miR-125a and ET mRNA/protein.

Previous studies showed that miR-125a is a key player in the development of DN via targeting IL-6R, and the decreased expression of miR-125a can promote the proliferation of mesangial cells by increasing the expression of IL-6R. Downregulation of miR-125a can be, at least partially, attributed to the presence of rs12976445 polymorphism, which compromises the maturing process of miR-125a, demonstrating that miR-125a can be used as a novel diagnostic and therapeutic target for DN^52^. It was also demonstrated that miR-125a expression is substantially decreased in the ERAF group and subjects genotyped as GG. Similarly, the expression of IL-6R is substantially increased in the ERAF group and subjects genotyped as GG. Moreover, 248 AF patients were recruited in a study to show that the patients harboring the GG genotype had a longer disease-free survival^52^.

The results obtained from our study are limited by the small sample size used, and addition animal experiments are also preferred to further validate the findings of this study. Therefore, in our future study, it is necessary to recruit more subjects and establish an appropriate animal model to further support our findings.

## Conclusion

Our results showed that miR-125a is a key player in the development of IA rupture via targeting ET1, and the reduced expression of miR-125a increases the risk of post-SAH re-bleeding by increasing the expression of ET1. The rs12976445 polymorphism can at least partially affect the expression of miR-125a by compromising its maturation, demonstrating that miR-125a may be used as a novel diagnostic and therapeutic target for the treatment of IA rupture.

## Supplementary Information

Supplementary Information.

## Data Availability

The data that support the findings of this study are available from the corresponding author upon reasonable request.
