# Supplementary material for: Rs12976445 polymorphism is associated with the risk of post-SAH re-bleeding by modulating the expression of microRNA-125 and ET-1
Source: Sci Rep. 2022 Feb 8;12:2062. doi: 10.1038/s41598-021-04330-4 (PMC8825803; doi:10.1038/s41598-021-04330-4)

Rs12976445 polymorphism is associated with the risk of post-SAH re-bleeding by modulating the expression of microRNA-125 and ET-1

Wenping Xiong [1], Weiqi Yao [1], Zeyuan Gao [1], Kui Liu [1*]

1. Department of Neurosurgery, Zhongnan Hospital of Wuhan University, Wuhan, China, 430071
2. Department of Hematology, Union Hospital, Huazhong University of Science and Technology; Hubei Engineering Research Center for Human Stem Cell Preparation and Application and Resource Conservation, Wuhan, China, 430022

Correspondence to: Kui Liu, Department of Neurosurgery, Zhongnan Hospital of Wuhan University, No.169 Donghu Road, Wuhan, China, 430071

Email: neurondrx@163.com

Western Blots

Original Image for Fig 5 ET1

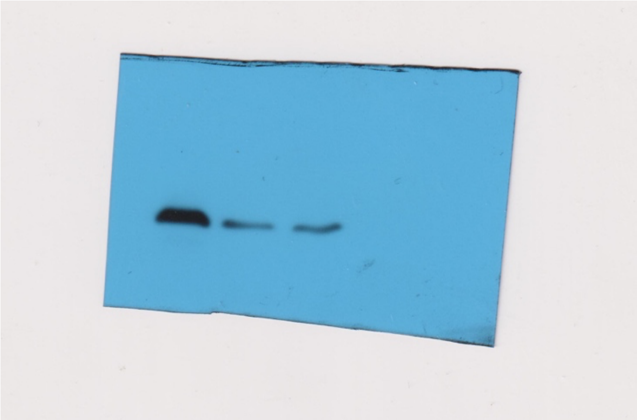

Original Image for Fig 5 beta actin

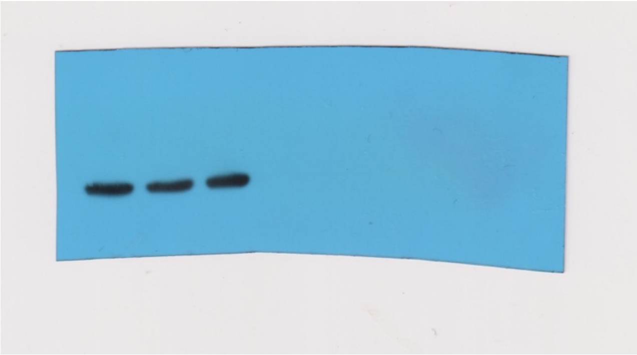

Original Image for Fig 6 ET1

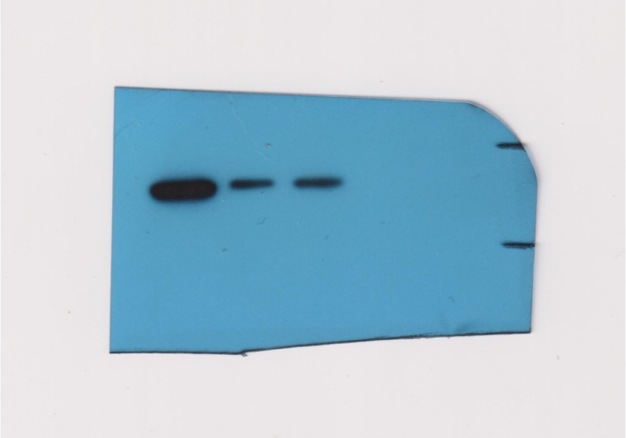

Original Image for Fig 6 beta actin

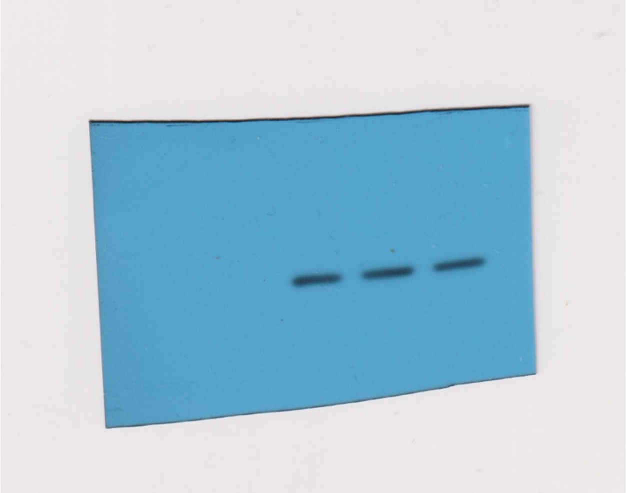

Supplement: Supplementary file 1 — Supplementary Information. [file 41598_2021_4330_MOESM1_ESM.pdf]
